# The CD40–CD40L Dyad in Experimental Autoimmune Encephalomyelitis and Multiple Sclerosis

**DOI:** 10.3389/fimmu.2017.01791

**Published:** 2017-12-12

**Authors:** Suzanne A. B. M. Aarts, Tom T. P. Seijkens, Koos J. F. van Dorst, Christine D. Dijkstra, Gijs Kooij, Esther Lutgens

**Affiliations:** ^1^Department of Medical Biochemistry, Subdivision of Experimental Vascular Biology, Academic Medical Center, University of Amsterdam, Amsterdam, Netherlands; ^2^Institute for Cardiovascular Prevention (IPEK), Ludwig Maximilians University (LMU), Munich, Germany; ^3^Medical Faculty, VU University Medical Center, Amsterdam, Netherlands; ^4^Department of Molecular Cell Biology and Immunology, Neuroscience Campus Amsterdam, VU University Medical Center, Amsterdam, Netherlands

**Keywords:** CD40, CD40L, multiple sclerosis, experimental autoimmune encephalomyelitis, inflammation, tumor necrosis factor receptor-associated factors

## Abstract

The CD40–CD40L dyad is an immune checkpoint regulator that promotes both innate and adaptive immune responses and has therefore an essential role in the development of inflammatory diseases, including multiple sclerosis (MS). In MS, CD40 and CD40L are expressed on immune cells present in blood and lymphoid organs, affected resident central nervous system (CNS) cells, and inflammatory cells that have infiltrated the CNS. CD40–CD40L interactions fuel the inflammatory response underlying MS, and both genetic deficiency and antibody-mediated inhibition of the CD40–CD40L dyad reduce disease severity in experimental autoimmune encephalomyelitis (EAE). Both proteins are therefore attractive therapeutic candidates to modulate aberrant inflammatory responses in MS. Here, we discuss the genetic, experimental and clinical studies on the role of CD40 and CD40L interactions in EAE and MS and we explore novel approaches to therapeutically target this dyad to combat neuroinflammatory diseases.

## Introduction

Multiple sclerosis (MS), a chronic inflammatory, demyelinating disease of the central nervous system (CNS), affects approximately 2.5 million people worldwide and is the most common cause of non-traumatic neurological disability in young adults (1). MS can be subdivided in different disease courses, including relapsing remitting MS (RRMS), secondary progressive MS (SPMS), and primary progressive MS (PPMS) (2). At disease onset, 85% of the patients have RRMS, which is characterized by acute attacks (relapses) followed by a period of partial or full recovery (remission) of the symptoms. Approximately 50% of these patients will subsequently develop SPMS. Although the etiology of MS is unknown, the disease is characterized by dynamic inflammatory lesions, consisting of activated T cells, B cells, macrophages and CNS-resident cells that eventually cause severe CNS tissue damage resulting in neurological deficits (3–6). Glucocorticoids are commonly used to inhibit the inflammatory response causing relapses. Although these drugs promote a faster recovery, there are no long-term neuroprotective effects (7, 8). In RRMS, reduced frequency of relapses and inhibition of disease progression is observed upon treatment with disease modifying drugs, including interferons, glatiramer acetate, sphingosine-1-phosphate receptor modulators and monoclonal antibodies directed against α4-integrin (natalizumab), CD52 (alemtuzumab), CD25 (daclizumab), and CD20 (ocrelizumab, ofatumumab) (7). These agents successfully extended the treatment strategies for RRMS, but disease modifying drugs lacked efficacy in progressive MS and may have potentially severe adverse effects including cytopenias, infectious diseases, and progressive multifocal leukoencephalopathy (9–12). Identification of additional therapeutic targets, especially for progressive MS, is therefore a widely recognized scientific goal with great clinical implications.

CD40 is a membrane-bound costimulatory protein and is a member of the tumor necrosis factor receptor (TNFR) family. CD40 is constitutively expressed by B cells and dendritic cells, but upon cell activation the protein is broadly expressed on hematopoietic cells, including T cells, monocytes and macrophages, but also on non-hematopoietic cells, such as endothelial cells (ECs) and CNS resident cells. The classical ligand for CD40 is the tumor necrosis factor (TNF) family member CD40 ligand (CD40L), which is expressed on both T cells and platelets. During inflammation CD40L is also expressed on B cells, dendritic cells, monocytes, macrophages, EC, and CNS resident cells, amongst others. CD40-mediated signaling depends on adaptor molecules, the TNF-receptor-associated factors (TRAFs) that bind to the cytoplasmic tail of CD40 and can activate multiple signaling cascades dependent on the TRAF family member that binds and the cell-type that is activated. The CD40 cytoplasmic domain has a proximal TRAF-6 binding site and a more distal TRAF-2/3/5 binding site (13). The CD40–CD40L dyad is an immune checkpoint regulator that promotes both humoral and cellular immune responses by regulating the inflammatory phenotype of immune and non-immune cells. Genetic and antibody-mediated inhibition of CD40 or CD40L successfully reduced disease burden in experimental models of atherosclerosis, Crohn’s disease, psoriasis, rheumatoid arthritis (RA), and experimental autoimmune encephalomyelitis (EAE) (14).

Experimental studies identified the CD40–CD40L dyad as a potent therapeutic target in MS (15–21). A pilot study with anti-CD40L mAb IDEC-131 in MS patients was successful, which led to the launch of a phase II trial. Unfortunately, this trial was halted after a case of severe thromboembolism in an IDEC-131 trial in Crohn’s disease patients (22). Clinical applicability of antibody-mediated blockage of CD40 is compromised by the risk of severe immunosuppression. Interestingly, recent insights in the downstream CD40 signaling pathways identified novel possibilities to inhibit the CD40–CD40L dyad without these side effects (13, 23).

In this review, we discuss genetic, experimental, and clinical studies on the role of CD40 and CD40L in the neuroinflammatory response underlying MS and we explore novel strategies that may eventually overcome the current limitations of antagonizing the CD40–CD40L dyad in MS.

## Expression of CD40L During MS

Our knowledge on the expression of CD40L and CD40 in MS is based on postmortal human studies and on reports from studying EAE, a widely used animal model of MS. In this model, neuroinflammation is initiated by T cells. However, for demyelination monocyte-derived macrophages, opsonizing antibodies and complement have been shown to play an essential role (24).

### T Cells

In order to initiate a proper T cell-mediated immune response, cell–cell interactions between T cells and antigen-presenting cells (APCs), such as B cells, macrophages, and dendritic cells are required. Three distinct signals are needed for T cell activation: binding of the T cell receptor with the MHC class II complex on APCs is the first signal, the second signal is generated by costimulatory molecules, and the third signal originates from cytokines. CD40L expressing T cells can activate resting APCs *via* interaction with their CD40 receptors. Upon activation the APCs will upregulate cytokine receptors and other costimulatory molecules (25).

**Figure 1:**
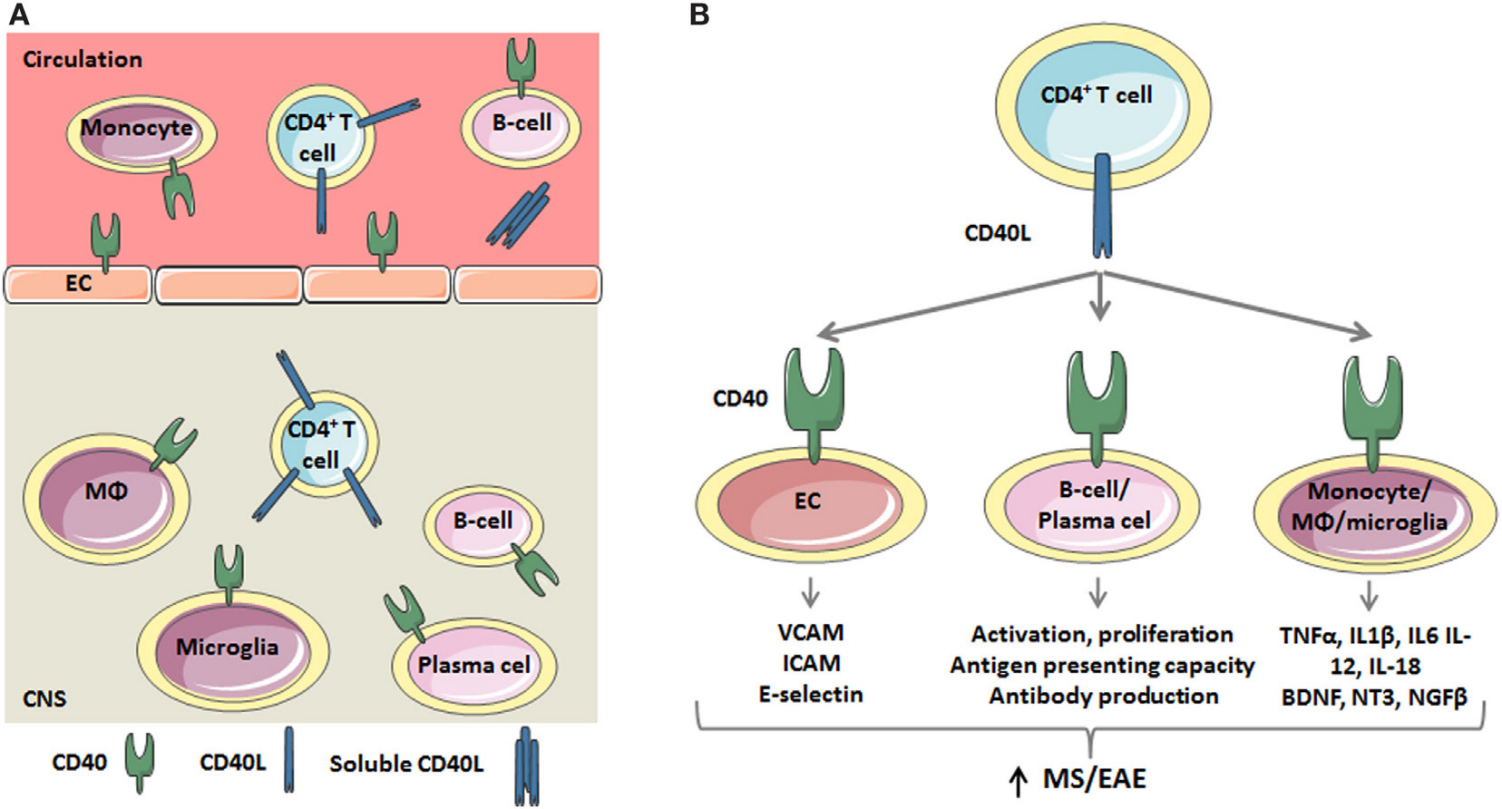
The critical role of the CD40 ligand (CD40L)–CD40 dyad in the inflammatory response underlying multiple sclerosis (MS)/experimental autoimmune encephalomyelitis (EAE). **(A)** During MS/EAE, the number of CD40L^+^CD4^+^ T cells in the peripheral blood and central nervous system (CNS) increases. Besides a membrane bound form, CD40L also exists as soluble trimer, which is mainly derived from platelets. CD40L interacts with CD40 on endothelial cells (ECs) and circulating monocytes and B cells. Within the CNS, T cells activate CD40^+^ macrophages, microglia, B cells, and plasma cells. **(B)** CD40L-mediated activation of CD40 on EC results in the expression of adhesion molecules, including VCAM, ICAM, and E-selectin, which promotes the recruitment of inflammatory cells to the CNS. CD40L also induces B cell activation, characterized by CD69 expression, and proliferation. Furthermore, the antigen presenting capacity of B cells is improved as a result of increased MHC class II, CD54, CD80, and CD86 expression. CD40L also promotes the secretion of proinflammatory cytokines by circulating monocytes and macrophages and microglia in the CNS. Thus, the CD40L–CD40 dyad critically regulates both adaptive and innate immune responses.

Both CD4^+^ and CD8^+^ T cells are abundantly present in MS lesions. During immune activation, both T cell subsets can express CD40L, however, in MS CD40L expression is only detected on CD4^+^ T cells, and not CD8^+^ T cells (26). CD40L is not detected in the healthy CNS, nor in the CNS of patients with other neurodegenerative disorders like Alzheimer’s Disease (15), suggesting that infiltrated CD40L^+^ T cells are the driver of CD40-mediated inflammation in MS. Infiltrated CD40L^+^ T cells induce activation of the various CD40-expressing cells (27) (Figure 1A). Likewise, in murine relapsing-remitting EAE, CD40L-expressing T cells infiltrate the CNS as early as day 4 postimmunization, and the number of CD40L^+^ T cells increased in the acute phase and peaked during remission, indeed suggesting that CD40L drives the initial phases of neuroinflammation (28).

### Soluble CD40L (sCD40L)

Besides membrane-bound CD40L, CD40L also exists as a soluble protein: sCD40L, which is mainly derived from activated platelets (95%) and T cells (5%) (29, 30). After cleavage from the platelet surface, sCD40L remains trimeric and can bind to integrin α_IIb_β_3_ on platelets or the CD40 receptor, which induces the expression of inflammatory mediators, such adhesion molecules, tissue factor, and chemokines (30).

Multiple population studies have demonstrated that serum sCD40L concentrations were increased in MS patients with active disease compared to healthy controls (5.65 ± 2.87 vs. 0.14 ± 0.12 ng/mL, *p* < 0.001) or patients with inactive MS (5.65 ± 2.87 vs. 0.64 ± 0.30 ng/mL, *p* < 0.001) (31, 32). A similar increase of sCD40L was detected in the CSF of MS patients compared to patients suffering from other inflammatory or neurological diseases (38.5 vs. 4.8 pg/mL, *p* < 0.002; SD not mentioned in the manuscript) (33). Although serum sCD40L concentrations, but not CSF sCD40L concentrations, positively correlated (Kendall tau-*b* = 0.29, *p* = 0.044) with the CSF/serum albumin ratio (“Qalb”), an indicator of blood-brain barrier permeability (33), it is currently unknown whether sCD40L directly contributes to BBB breakdown. Interestingly, serum concentrations of sCD40L decreased upon treatment with Glatiramer acetate (copaxone) (34), IFN-β (35), or natalizumab (36), suggesting that sCD40L can be used as a biomarker to monitor the effectiveness of these therapies.

In contrast to these results, a large cohort study with 833 MS patients showed decreased levels of sCD40L in patients with inactive MS compared to healthy individuals (86.3 ± 9.3 vs. 54.3 ± 5.4 pg/mL, *p* < 0.001) (37). Several factors may contribute to these conflicting findings, including the use of antiplatelet drugs, such as cyclooxygenase inhibitors or adenosine diphosphate receptor inhibitors, which limit the release of sCD40L from activated platelets (29). Importantly, the use of these agents has not been reported in the population studies. In addition, a circadian rhythm in serum sCD40L levels has been observed in patient suffering from myocardial infarction, sCD40L levels were 41.5% higher in samples obtained at 9 p.m. compared to samples drawn at 2 a.m., possibly due to diurnal fluctuations in proteinase levels (38). Whether a similar circadian rhythm is present in MS patients is currently unknown. Finally, differences in blood sample handling may affect serum sCD40L levels, as low temperatures limit the *ex vivo* release of sCD40L from platelets (29). Carefully monitoring of these factors in future studies is required to fully elucidate the role of sCD40L in MS.

## Expression of CD40 During MS

### Macrophages and Microglia

Autopsy studies in MS patients revealed that monocytes, macrophages and activated microglia are the main cell types expressing CD40 in the CNS (15). Microglia in a resting state show low or no CD40 expression, while ~45% of the activated microglia and ~73% of recruited peripheral macrophages express CD40 during EAE (39). Macrophages form a functionally heterogeneous population, with proinflammatory M1 macrophages and anti-inflammatory M2 macrophages representing the extremes of a spectrum that is present *in vivo* (40). CD40 is an M1 marker for perivascular macrophages, activated microglia and myelin-loaded macrophages in MS lesions and its expression is associated with the coexpression of other M1-markers, such as CD86, CD64, and CD32. CD40L-induced activation of these cells results in the secretion of M1-associated cytokines and chemokines, including interleukin (IL)-1, IL-6, IL-12, IL-18, and TNF-α (41–43), which fuels the ongoing inflammation in the CNS (Figure 1B) (44–47). However, 70% of the CD40^+^ cells also express M2 markers, including CD163 and CD206, suggesting that a mixed M1/M2 phenotype exists in MS lesions (48). The abundant expression of CD40 on mononuclear cells in perivascular infiltrates was also found in the brain of marmoset monkeys (*Callithrix jacchus*) with acute EAE (24). In murine relapsing-remitting EAE, CD40 expressing cells infiltrate the CNS as early as day 4 postimmunization and the numbers of CD40^+^ cells peaked in the acute and relapsing phases of the disease and decreased during remission (28). The amount of CD40 present in the CNS correlated with the expression of the inflammatory cytokines IL-12, IFN-γ, and TNF-α (28). Correspondingly, in the mouse spinal cord CD40 was abundantly expressed by monocytes and monocyte-derived macrophages (17). The expression of CD40 in the CNS found in mice and marmoset monkeys suggests that there is HLA class II-restricted antigen presentation, and that effector functions of CD40 expressing macrophages are triggered by CD40L-expressing activated CD4^+^ T cells (17, 24). *In vitro*, interactions between T cells and macrophages can stimulate the production of proinflammatory cytokines, nitric oxide, and matrix metalloproteinases, which are all components that play a role in the immunopathogenesis of MS and other chronic inflammatory diseases (17).

### B Cells

In order to initiate a humoral immune response, CD40L present on activated CD4^+^ T cells needs to interact with the CD40 receptor on antigen activated B cells. During this response, high titers of isotype-switched, high affinity antibodies are generated by the B cells and germinal centers (GCs) are formed (49, 50). Furthermore, CD40–CD40L signaling between follicular helper CD4^+^ T cells (T_FH_) and GC B cells is required for the formation of memory B cells, antibody-secreting plasma cells and the maintenance of GC (51, 52). The number of circulating CD40^+^CD20^+^ B cells did not differ between MS patients and controls; however, the density of CD40 on these cells was increased, which may have several effects on B cell function (53). First, CD40 activation on both naïve (CD19^+^CD27^−^) B cells and memory (CD19^+^CD27^+^) B cells results in the activation of these cells, characterized by CD69 expression (54). Second, the proliferative response of both naïve and memory B cells is increased upon CD40 activation (55). Third, the antigen presenting capacity of B cells was also affected as upon ligation of CD40 by CD40L, B cells from MS patients exhibited increased expression of MHC class I and II, CD54, CD80 and CD86, which promoted the activation and proliferation of T cells, especially CD4^+^ T cells. These CD4^+^ T cells subsequently induced the proliferation of CNS-antigen specific CD8^+^ T cells, at least *in vitro* (54, 56).

NF-κB and MAPKs (P38, ERK, and JNK) are essential components of signaling pathways downstream of CD40 binding in B cells. Following CD40 stimulation, memory and naive B cells from MS patients showed a significantly higher level of NF-κB activation, reflected by increased levels phosphorylated p65, compared with healthy controls (57). Treatment with glatiramer acetate (Copaxone) reduced the phosphorylation of p65 in B cells of RRMS patients to levels observed in healthy individuals. These results suggest that reducing CD40-mediated activation of the canonical NFkB pathways may be a common mechanism by which some existing treatments limit inflammation in MS (57).

In recent years, a novel subset of regulatory B cells, which secrete anti-inflammatory cytokines such as IL-10 and TGF-β, has been described (58). CD40-induced activation of these cells reduced inflammation and disease severity in a murine model of systemic lupus erythematosus in an IL-10-dependent manner (59). However, Michel et al. reported that both the frequency and function of regulatory B cells was not affected in MS patients, specifically, CD40L-induced cytokine secretion was unaffected, suggesting that a similar mechanism is not present in MS (60).

The clinical success of B cell depleting therapies implicates an important role for these cells in the pathogenesis of MS. Indeed, ectopic B cell follicles have been detected in the meninges of patients with progressive MS, immunoglobulin depositions are present in MS lesions and oligoclonal immunoglobulins are detected in the CSF of 90% of the patients (61). Although the pathologic effect of these autoantibodies is incompletely understood, several autoantigens have been detected, including MOG, neurofascin, sperm-associated antigen 16 (SPAG16), contactin-2 and inward-Rectifying Potassium Channel (KIR4.1) (62). Interestingly, the CD40–CD40L dyad has a critical role in B cell biology, as it regulates antibody production, immunoglobulin isotype switching and B cell follicle formation, accordingly, antibody-mediated inhibition of CD40 reduced anti-MOG antibody production in non-human primates subjected to EAE (20). Whether CD40 signaling plays a relevant role in the generation of autoantibodies in the context of MS is currently unknown.

### CD40 Expression on T Cells

CD40 expression is mostly described on APCs, but CD4^+^ and CD8^+^ T cells can also express low levels of CD40 mRNA that increase after activation. Effector T cells deficient for CD40 have poor capacity to proliferate, and antigen stimulated cytokine secretion during the primary immune response and in the memory phase is as low as that of naive T cells. Priming with CD4^+^ T cells did not increase the proliferation and cytokine secretion capacity of CD40-deficient T cells, demonstrating that CD4 help to CD8^+^ T cells requires interactions with CD40 expressed by CD8^+^ T cells (63). The role of CD8^+^ T cells in MS and EAE is not completely understood and is rather controversial with evidence for both a pathogenic and a regulatory function (64). The role of CD4^+^ T cells expressing CD40 (CD40^+^CD4^+^ T cells) in EAE is recently investigated. CD40^+^CD4^+^ T cells are found in lesions in the CNS and stimulate a more severe EAE disease development than conventional CD4^+^ T cells (65). Adoptive CD40^+^CD4^+^ T cell transfer from EAE-induced donors transfers EAE without further *in vitro* expansion and without requirement of an EAE inducing procedure to the recipient animals. Coinjection of the CD40^+^CD4^+^ donor T cells with CFA in the recipient animals results in a more severe disease outcome (65), indicating that in addition to the specific T cell response to CNS antigen, a general activation of the immune system is necessary to induce severe disease. Moreover, this suggests that any event leading to CD40^+^CD4^+^ T cell expansion might result in susceptible individuals.

### Endothelial Cells

In addition to immune cells, 25% of brain EC constitutively express CD40 *in situ* and *in vitro* and the expression is further increased by inflammatory stimuli (48, 66–68). Activation of endothelial CD40 by CD40L induces the expression of the adhesion molecules E-selectin, VCAM-1, and ICAM-1 (Figure 1B), which facilitates the migration of monocytes and CD4^+^ T and CD8^+^ T cells into the CNS. Brain ECs also induce T cell activation and proliferation *via* MHC-II-dependent antigen presentation and CD40-mediated costimulation (66). Thus, CD40–CD40L-mediated interactions between EC and immune cells promote CNS inflammation by facilitating transmigration of leukocytes across the BBB and subsequently inducing T cell activation and proliferation (67).

### Resident CNS Cells

CD40 is also expressed on other resident CNS cell types like astrocytes and neurons. Astrocyte CD40 induces the secretion of proinflammatory cytokines and chemokines, which trigger an autocrine activation of these cells that aggravate EAE (69). CD40 is constitutively expressed on neurons (70). CD40L-induced activation of primary cultured neuronal cells results in activation of p44/42 MAPK signaling pathways, and increases neurofilament expression, a marker of neuronal differentiation. In addition, CD40 has a critical role in neuronal survival as neuronal cell injury induced by serum withdrawal can be rescued by CD40 ligation in wild-type neurons, but injury could not be reduced by CD40 ligation in CD40-deficient neurons. These *in vitro* findings are further supported by observations in aged *Cd40*^−/−^ mice. Examination of the brain of CD40-deficient mice showed that at older age (16 months) CD40 deficiency results in decreased neurofilament expression, neuronal dysmorphology, reduced brain weights, and increased TUNEL reaction, indicating increased presence of apoptotic cells (70). Additionally, phenotypic analysis of *Cd40*^−/−^ mice showed that CD40 regulates growth from excitatory and inhibitory neuron dendrites (71). In conclusion, neuronal CD40 has an important role in neuronal development, maintenance, and survival.

### Expression of CD40–CD40L on Circulating Immune Cells in MS

In addition to the local alterations in the CNS discussed above, the systemic immune system is also affected in MS (1). The expression of CD40 and CD40L on peripheral blood monocytes, especially the CD16^+^ proinflammatory subset, is increased in MS patients, reflecting the higher activation status of these cells (53, 72–74). Besides inflammatory cytokines, monocytes also produce protective anti-inflammatory mediators upon CD40L-induced activation such as the neurotrophins BDNF, NT3, and NGFβ (39). Interestingly, this protective response is reduced in monocytes from MS patients, but restored upon IFNβ treatment (75–77).

Circulating CD40L^+^ CD4^+^ T cells and CD40L^+^ CD8^+^ T cells are also more abundant in MS patients compared to healthy controls (53, 78). Upon *in vitro* CD3-induced reactivation, CD4^+^ T cells from MS patients expressed more CD40L and produced increased levels of inflammatory mediators, compared to T cells from healthy controls, suggesting that CD40L is especially expressed by activated proinflammatory T cells (79). Interestingly, IFNβ reversed the increased expression of CD40L on CD4^+^ T cells (53, 80).

Taken together, during neuroinflammation, CD40L^+^ CD4^+^ T cells infiltrate the CNS and activate CD40^+^ monocytes, macrophages, B cells, ECs, and other CNS resident cells, which propagates the ongoing inflammatory response and aggravates lesion development (Figures 1A,B). Consequently, two strategies can be applied to therapeutically target the CD40–CD40L dyad in MS; (1) inhibition of CD40 on resident and immune cells; (2) inhibition of CD40L on T cells. While the role of CD40L-induced activation of CD40^+^ cells in immunity and inflammation is well known, only limited data exist on the reciprocal activation of CD40L^+^ cells. The intracellular domain of CD40L does not contain any signaling motifs, however upon activation its transmembrane domain associates with lipid rafts, thereby inducing AKT and p38 MAPKs pathways and the subsequent production of IL-2, based on *in vitro* experiments (81). Additionally, this may promote IL-4, IL-10, TNF-α, and IFN-γ production (82). However, the pathophysiological relevance of the reciprocal activation of CD40L^+^ cells in MS or other inflammatory diseases has not been explored and requires further attention as it may result in the identification of novel therapeutic targets.

## CD40 Single-Nucleotide Polymorphisms (SNPs) and MS

Genome-wide association studies have identified a correlation between SNPs in immune related loci, including the CD40 locus, and the incidence of MS (83). In particular, the SNP rs1883832C- > T in the CD40 gene was associated with an increased risk for MS. Compared to rs1883832CC individuals, heterozygous rs1883832CT and homozygous rs1883832TT individuals had a 1.5-fold and 2.5-fold increased risk for MS, respectively (84). Counterintuitively, the high-risk allele was associated with a 45.5% decreased expression of CD40 mRNA in an *in vitro* translation/transcription system (85, 86). A second CD40 SNP (rs6074022T- > C) was also associated with a minor decrease in the expression of CD40 mRNA in whole blood RNA from MS patients (87–89). Thus, at least two high risk SNPs are associated with a decreased expression of CD40, which seems contradictory and requires further attention, as *in vivo* studies have established the proinflammatory role of CD40 in neuroinflammation, as discussed below. In RA and Graves’ disease CD40 rs1883832 is associated with reduced CD40 expression and disease protection. These findings are in line with the role of CD40 as a costimulator in T cell activation supporting the autoimmune inflammatory process (86). How to explain the increased risk for MS upon decreased CD40 mRNA expression is so far unclear. Until now, no polymorphisms of the CD40L gene have been associated with MS (84).

## Promising Therapeutic Potential of the CD40L–CD40 Dyad for EAE and MS

Several EAE studies in mice and non-human primates showed that the CD40–CD40L signaling pathway is an interesting target to reduce incidence and severity of neuroinflammation. Combination therapies can even further increase treatment efficiency. A discussion of this research is described here.

### Genetic Deficiency of CD40 or CD40L in Mouse EAE

Both CD40- and CD40L-knock-out mice do not develop EAE after immunization. CD40–CD40L interactions are required for the B7.1 and B7.2 expression on APCs, essential for T cell activation. Lack of costimulation through B7.1 and B7.2 may result in a reduction of secondary signaling and prevention of T cell activation, possibly responsible for protection of CD40L-deficient mice from EAE (16). Experiments with *Cd40*^−/−^ mice showed that in the absence of CD40, T cells both enter the CNS and induce disease. This suggests that activated T cell trans-migration through the endothelial BBB does not require CD40 (90). However, CD40 is necessary for Th1 cell activation as in the presence of CD40, there is an earlier entrance of Th1 cells into the CNS and more severe induction of disease (90). CD40 on peripheral hematopoietic cells is known to be pivotal to the development of autoimmunity (25). By using bone marrow (BM) chimeric mice, Becher et al. showed that lack of expression of CD40 on CNS-resident microglia also diminishes EAE severity and reduces the amount of leukocyte infiltration into the CNS (91). Reduced microglial expression of CD40 did not affect peripheral T cell priming or recall responses. Encephalitogenic T cells could not elicit the expression of chemokines in a CNS environment in which parenchymal microglia were CD40 depleted. So, outside of the systemic immune compartment CD40 increases organ-specific autoimmunity and within the CNS CD40 expressing cells regulate the EAE development in BM chimeric mice (91).

### Antibody-Mediated Inhibition of CD40L in Mouse EAE

Treatment of EAE-induced mice with an antagonistic anti-CD40L mAb (MR1) during disease induction (days 0–4) completely prevented development of disease (15). Treatment during days 4–8 and days 7–11 after induction reduced disease burden by 80 and 67%, respectively (15). Antibody treatment inhibited CNS inflammatory processes, as the number and size of CNS infiltrates of animals treated with anti-CD40L antibodies during EAE induction were strongly reduced compared to animals treated with an irrelevant antibody (17). Besides treatment at disease induction, EAE disease development and CNS inflammation were also blocked effectively by anti-CD40L antibody treatment of animals at the peak of acute disease and by treatment during remission (18). Interestingly, transient anti-CD40L blockade at the peak of the acute phase of R-EAE in SJL mice reduced clinical relapses by 80%, and in mice that did develop a relapse, the duration and severity was reduced as compared to control antibody treated animals (19). Short-term (4 days) treatment with anti-CD40L during EAE induction could prevent clinical disease and did not affect the long-term Th1/Th2 balance (92).

### Mechanism of Disease Reduction upon Anti-CD40L Treatment

**Figure 2:**
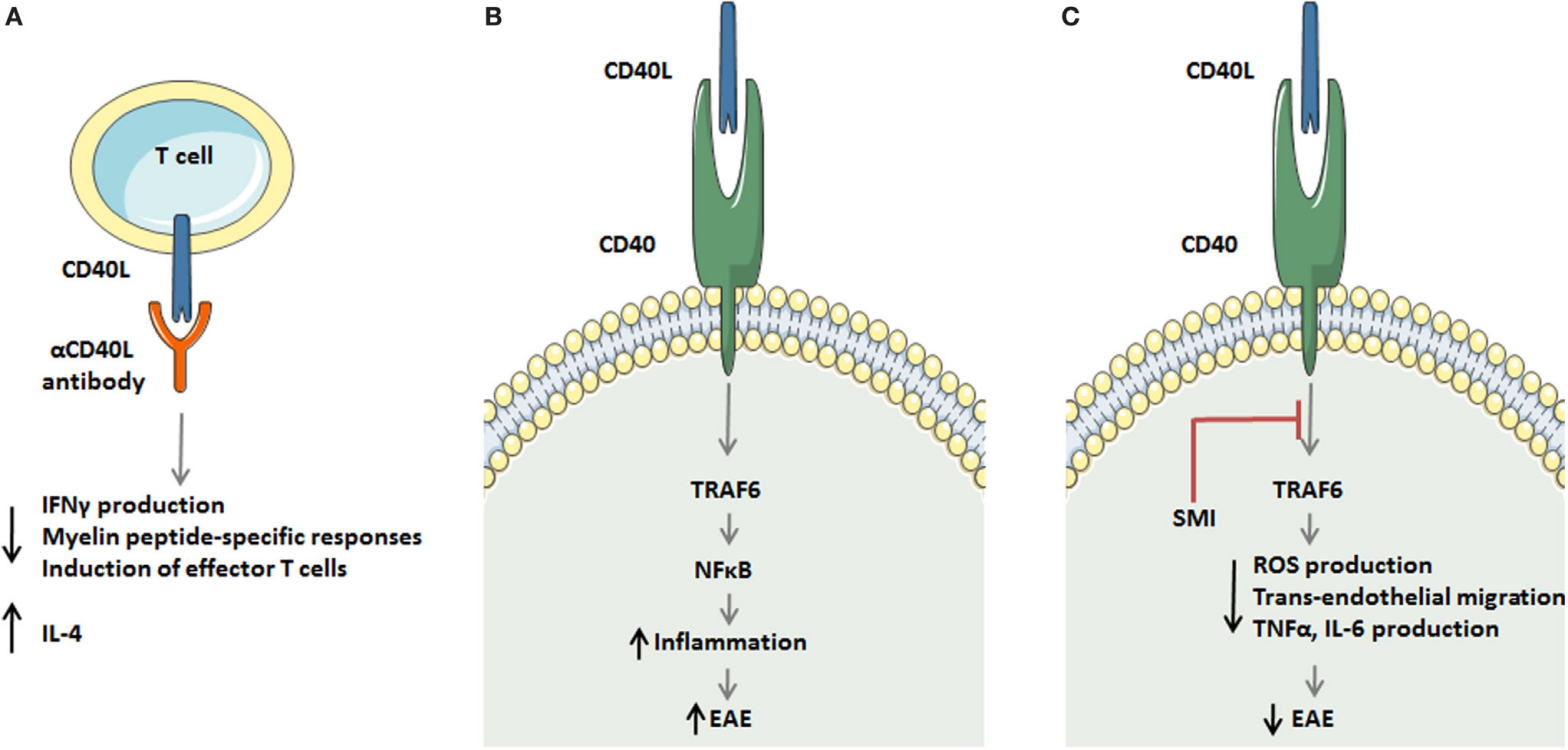
Strategies to target the CD40 ligand (CD40L)–CD40L dyad in experimental autoimmune encephalomyelitis (EAE). **(A)** Antagonistic CD40L antibodies limit detrimental T cell responses in multiple sclerosis (MS) by decreasing IFNγ production, reducing myelin peptide-specific delayed-type hypersensitivity responses, and limiting in effector T cell formation. In addition, antibody treatment enhances the production of the Th2 cytokine interleukin (IL)-4. **(B,C)** A novel strategy to target the CD40L–CD40 axis in monocytes and macrophages during EAE. The CD40L–CD40–TRAF6 axis activates the transcription factor NF-κB, which subsequently induces an inflammatory monocyte/macrophage phenotype that aggravates EAE. Small molecule-mediated inhibition of the CD40–TRAF6 interaction reduces ROS, TNFα, and IL6 production by macrophages and limits transendothelial migration of monocytes, thereby improving EAE in mice and rats.

Myelin-specific T cells in anti-CD40L-treated mice subjected to EAE secreted little IFNγ but exhibited strongly enhanced levels of IL-4, compared to control treated mice (Figure 2A). Anti-CD40L mAb did not result in systemic tolerance of encephalitogenic T cells (93) nor caused expansion of myelin-specific T cells. However, treatment with anti-CD40L antibody at the peak of acute disease or during remission inhibited Th1 differentiation and effector function. While T cell proliferation and secretion of the cytokines IL-2, IL-4, IL-5, and IL-10 were normal (18), antibody treatment strongly impaired IFN-γ production, delayed-type hypersensitivity responses against myelin peptide, and encephalitogenic effector cell activation (18, 93). Treatment with anti-CD40L antibody also reduced clinical disease expression in adoptive recipients of encephalitogenic T cells, suggesting involvement of CD40–CD40L interactions in their effector ability to activate CNS macrophages/microglia (18).

CD40–CD40L interactions between APCs and T cells results in IL-12 secretion, an essential cytokine for Th1 responses and EAE induction. This has led to the hypothesis that the protective effects of CD40L blockade in EAE can be overcome by IL-12 administration. Mice cotreated with exogenous IL-12 and the anti-CD40L antibody developed severe EAE and anti-IL-12 antibody treatment protected mice from EAE (94). However, the protein IL-12 consists of a p35 and a p40 subunit, the IL-12p40 subunit is shared by other cytokines. *Il-12p40*^−/−^ mice are protected against EAE, but *Il-12p35*^−/−^ mice develop severe EAE, verifying that the p40 subunit, and not IL-12 is critical for the development of EAE (95). IL-23, another cytokine containing the p40 subunit, has later been shown to play a critical role in the EAE disease development instead of IL-12 (96, 97). Whether the beneficial effect of CD40 blockade in EAE is mediated through reduced levels of IL-23 is not yet investigated, and the role of CD40–CD40L interactions in IL-23 secretion is still unclear.

To investigate whether the mechanism of EAE inhibition by anti-CD40L mAb depends on its Fc effector interactions, Nagelkerken et al. compared an anti-CD40L mAb (produced in mammalian cells) with its a-glycosylated counterpart, which has strongly reduced FcγR binding and impaired complement binding activity. They found that both forms of the Ab have similar ability to inhibit clinical signs of EAE (98). Therefore, in the context of EAE, FcR interactions do not play a crucial role in the protective effect of anti-CD40L mAb (98). Activation of microglial cells is a multistep process and microglial cell CD40 expression facilitates EAE disease development. Activation of microglial cells at the onset of EAE is a process independent of CD40, and their activation is characterized by increased expression of CD45 and MHC class II. However, at the peak of disease, complete activation of microglial cells is dependent on their CD40 expression (39). These results show that in order to facilitate the progression of EAE clinical disease activation of microglial cells in the CNS is needed (39).

### Anti-CD40 and CD40L Antibody Treatment in EAE in Non-Human Primates

As treatment of EAE-induced mice with anti-CD40L mAb effectively blocked clinical disease progression and CNS inflammation (15, 17, 18), CD40L–CD40 interaction inhibiting experiments were extrapolated to non-human primate models of EAE. Anti-CD40 mAb showed beneficial activities in a EAE model in non-human primates when administered early in disease development (20) or after the onset of neuroinflammation (21). Inhibition of CD40–CD40L interactions was tested in marmoset monkeys with anti-human CD40 mAb (ch5D12), a chimeric antagonist. Severe clinical signs of EAE were observed in all placebo-treated monkeys, whereas in the ch5D12-treated group the animals did not develop disease symptoms at all. Postmortem analysis of the CNS showed that ch5D12 treatment resulted in a reduced lesion load (20). The same model was used to test the anti-human CD40 monoclonal antibody in its parent murine form (mu5D12). mu5D12 mAb treatment interfered with development of clinical symptoms, even when mu5D12 mAb was given several weeks after T cell priming. *In vivo* localization shows that in addition to entering the secondary lymphoid organs, the mu5D12 mAb also enters the perivascular spaces of the CNS and to a smaller extent penetrates in the brain parenchyma. Therefore, anti-CD40 can inhibit activation of primary and secondary antigen-specific T cells and B cells in both the secondary lymphoid organs and CNS lesions. The activity in secondary lymphoid organs is important since pathogenic T cells are continuously activated in the periphery during established EAE (21). Supporting this data, serial MRI demonstrated that ch5D12 treatment prevented the expansion of existing white matter lesions (99).

### Combination Therapy of CD40–CD40L Blockade and Other Disease Modifying Agents

Blocking CD40–CD40L has been proven to be effective in reducing EAE, as described above. Disruption of CD40–CD40L interaction blocks activation of autoantigen-specific T cells and decreased leukocyte infiltration into the CNS. Therefore, it would be interesting to restore self-tolerance by enhancing Tregs in addition to the inhibition of the proinflammatory mechanisms. To achieve this, we should block multiple costimulatory dyads simultaneously to further increase the efficiency of the treatment. In the oncology field it has already been shown that immune checkpoint inhibitor combination therapy results in better outcomes compared to the use of a single immune checkpoint inhibitor, an example is the combination of nivolumab (anti-PD-1) and ipilimumab (anti-CTLA-4) (100).

Inflammatory cytokine production by astrocytes is induced upon activation by the CD40–CD40L interaction in a mast cell coculture. These cytokines re-activate astrocytes leading to increased release of cytokines that contribute to aggravating EAE development. Pretreatment with a combination of anti-CD40 antibody and the Rac inhibitor 8-hydroxydeoxyguanosine (8-oxo-dG) decreased the EAE induced TNFR1 expression and colocalization of TNFR1 and astrocytes in the brain. This combination therapy was able to decrease the clinical scores further than one of the treatments alone (69). Moreover, analysis of EAE brain tissues show that anti-CD40 Ab and 8-oxo-dG treatment enhanced the number of Treg cells, increased OX40 expression, and increased production of cytokines associated with Treg cells and their suppressive function (101).

Treatment of EAE with a combination of the CD40L antagonistic MR1 antibody and CTLA4Ig, blocking the CD28-B7 interaction, provides additive protection in mice compared to single treatment, particularly in case of delayed administration. After treatment with the Anti-CD40L and CTLA4Ig combination mononuclear cell infiltrates were absent in the CNS, and in lymph nodes combination treatment is associated with a strong reduction in proliferation of primed T cells. According to these results, CD28 and CD40L might deliver different costimulatory signals for complete T cell activation, although there probably is an additional regulatory role for CD40L–CD40 interaction on B7 expression. Blocking both the CD40–CD40L and the CD28-B7 pathways *in vivo* possibly results in better suppression of pathogenic immune responses (102).

Simultaneous knock-down of CD40 and the p19 subunit of IL-23 expression on bone marrow-derived DCs (BMDCs) by injection of double-transduced CD40^+^p19LV^−^ BMDCs in EAE mice resulted in reduced clinical scores, significant decreased production of IL-17 and an increase in IL-10 compared with EAE mice treated with control lentiviral vector-DCs-(p19LV-DCs and CD40LV-DCs) (103). These studies show that combination therapies including blocking of CD40–CD40L interactions and an additional blockade can, by different mechanisms, more efficiently reduce EAE disease severity than anti-CD40L Ab or anti-CD40 Ab treatment alone.

### Anti-CD40L Treatment in Clinical Trials

The experimental studies in rodents and primates highlighted the therapeutic potential of CD40–CD40L targeting strategies in MS and (15–21) paved the way for clinical studies. Anti-CD40L mAb IDEC-131 treatment was found to be successful in a phase I clinical trial with 15 MS patients, in this trial no relapses were observed in the complete cohort for at least 6 months. After this pilot, a phase II clinical trial with 46 MS patients was initiated in 2002. However, due to a severe case of thromboembolism in a similar trial with anti-CD40L mAb in Crohn’s disease patients (22), these trials were halted. The thromboembolic complications were caused by disruption of CD40L-α_IIb_β_3_ interactions between platelets in arterial thrombi (13, 104, 105). For this reason, but also because of potential immunosuppressive adverse effects of antibody-mediated inhibition of CD40–CD40L, alternative strategies are required to exploit the therapeutic potential of CD40–CD40L inhibition in MS.

## Novel Strategies to Target the CD40–CD40L Dyad

One approach to reduce side-effects in treatment of EAE is specific delivery of drugs to CD40L positive T cells. Based on CD40L crystal structure and molecular docking studies, Ding et al. designed a CD40L specific peptide ligand (A25). The peptide A25 was conjugated on the surface of liposomes and capable of facilitating specific liposomal drug delivery to CD40L^+^ cells. CD40L^+^ cell ratios in EAE mice were significantly reduced by the A25 modified liposome loaded with methotrexate (MTX), a cytostatic drug, resulting in markedly reduced clinical scores (106).

Based on our recent findings, we propose that more specific downstream inhibition of the CD40L–CD40 dyad may be another approach to overcome the current limitations. Using mice specifically lacking CD40-TRAF6 or CD40-TRAF2/3/5 interactions, we showed that only CD40-TRAF6-deficient mice had a skewing in the immune response toward an anti-inflammatory profile and were protected against atherosclerosis (107). Based on these results, we developed small molecule inhibitors (SMIs) that efficiently and specifically block CD40-TRAF6 interactions and leave CD40-TRAF2/3/5 interactions intact (108). These SMIs are able to reduce peritonitis, sepsis, obesity-associated adipose tissue inflammation, and diabetes (108–111). Using an *in vitro* model for (neuro-) inflammation, we were able to show that SMI 6877002 skews the phenotype of human monocytes toward a less inflammatory profile with reduced monocyte trans-endothelial migration capacity across brain ECs *in vitro* (23). Furthermore, upon SMI treatment EAE disease severity was reduced in Lewis rats, but not mice. However, in both models the SMI-treated animals had reduced levels of CNS-infiltrated monocyte-derived macrophages, but not T cells (23). The experiments with SMI 6877002 in EAE illustrate the therapeutic potential of CD40-TRAF6 targeting strategies (Figures 2B,C), with the ability to reduce monocyte recruitment and macrophage activation in the CNS and this approach could potentially be used as a cotreatment to ameliorate MS.

## Conclusion and Future Directions

This review emphasizes that besides the classical adaptive immunity-related CD40L–CD40 signaling, this dyad has an essential role in the establishment and pathogenesis of MS in multiple ways. In particular, CD40 and CD40L are widely expressed on both resident and CNS-infiltrated cells in MS lesions and CD40 gene SNPs associate with MS incidence. There are several treatments available for RRMS, but additional therapeutic targets are still necessary, especially for progressive MS. Progressive MS is characterized more by nerve degeneration rather than inflammation. Monocyte-derived macrophages play an essential role in demyelination. In MS patients increased expression of CD40 on peripheral monocytes, and high expression of CD40 on myelin-loaded macrophages in MS lesions was observed. Using EAE as a model for MS, inhibition of the CD40–CD40L dyad has found to be an effective strategy to reduce the onset and development of EAE in rodent and primates. However, treatment with anti-CD40/CD40L mAb resulted in unforeseen thromboembolic side effects in human clinical trials, and bears the risk of immune suppression, which hampered further development of this strategy. Nevertheless, novel insights regarding treatment with combinations of immune checkpoint regulators, the use of nanoparticles, and the pivotal CD40L–CD40–TRAF signaling pathway in inflammatory diseases have revived the therapeutic potential of the CD40–CD40L dyad. These novel approaches are examples that the current limitations of long-term CD40 and CD40L inhibition in MS and other inflammatory diseases can be overcome. CD40–CD40L plays an important role in the pathogenesis of MS and research has proven that this dyad is an important therapeutic target for treatment of MS.

## Author Contributions

The study presented here was carried out in collaboration among all authors. All authors read and approved the final manuscript.

## Conflict of Interest Statement

The authors declare that the research was conducted in the absence of any commercial or financial relationships that could be construed as a potential conflict of interest.
